# Alkaloids from Marine Fungi: Promising Antimicrobials

**DOI:** 10.3390/antibiotics9060340

**Published:** 2020-06-18

**Authors:** Thomas Willems, Maarten L. De Mol, Aleksandar De Bruycker, Sofie L. De Maeseneire, Wim K. Soetaert

**Affiliations:** Centre for Industrial Biotechnology and Biocatalysis (InBio.be), Department of Biotechnology, Faculty of Bioscience Engineering, Ghent University, Coupure Links 653, 9000 Ghent, Belgium; Thomas.Willems@UGent.be (T.W.); Maarten.DeMol@Ugent.be (M.L.D.M.); Aleksandar.DeBruycker@UGent.be (A.D.B.); wim.soetaert@UGent.be (W.K.S.)

**Keywords:** antimicrobial compounds, marine fungi, alkaloids, isolation, biosynthesis, structure–activity

## Abstract

Resistance of pathogenic microorganisms against antimicrobials is a major threat to contemporary human society. It necessitates a perpetual influx of novel antimicrobial compounds. More specifically, Gram^−^ pathogens emerged as the most exigent danger. In our continuing quest to search for novel antimicrobial molecules, alkaloids from marine fungi show great promise. However, current reports of such newly discovered alkaloids are often limited to cytotoxicity studies and, moreover, neglect to discuss the enigma of their biosynthesis. Yet, the latter is often a prerequisite to make them available through sufficiently efficient processes. This review aims to summarize novel alkaloids with promising antimicrobial properties discovered in the past five years and produced by marine fungi. Several discovery strategies are summarized, and knowledge gaps in biochemical production routes are identified. Finally, links between the structure of the newly discovered molecules and their activity are proposed. Since 2015, a total of 35 new antimicrobial alkaloids from marine fungi were identified, of which 22 showed an antibacterial activity against Gram^−^ microorganisms. Eight of them can be classified as narrow-spectrum Gram^−^ antibiotics. Despite this promising ratio of novel alkaloids active against Gram^−^ microorganisms, the number of newly discovered antimicrobial alkaloids is low, due to the narrow spectrum of discovery protocols that are used and the fact that antimicrobial properties of newly discovered alkaloids are barely characterized. Alternatives are proposed in this review. In conclusion, this review summarizes novel findings on antimicrobial alkaloids from marine fungi, shows their potential as promising therapeutic candidates, and hints on how to further improve this potential.

## 1. Introduction

In recent years, multidrug resistance is becoming a major healthcare threat. Not only is the replenishment of the cornucopia of antimicrobial compounds approved to combat human infections steadily diminishing since the golden era (1940s–1970s) [1], microbial pathogens are also developing their own arsenal against these drugs. For example, carbapenem-resistant *Klebsiella pneumoniae*, fluoroquinolone-resistant *Escherichia coli*, cephalosporin-resistant *Neisseria gonorrhea*, methicillin-resistant *Staphylococcus aureus* (MRSA), and even colistin- and carbapenem-resistant *Enterobacteriaceae* were reported by the World Health Organization in many countries across the globe [2]. Due to their intrinsic toughness and the consequential lack of newly discovered antimicrobial drug leads, Gram^−^ bacteria are currently posing the biggest threat [3]. As last-resort antibiotics are starting to falter, an influx of novel antimicrobial compounds is necessary.

Filamentous fungi in general contribute approximately 20% to our antibiotic molecule library [4]. Moreover, marine-derived fungi hold an enormous potential of bioactive secondary metabolites [5,6,7]. Earlier, we reported on new antibacterial compounds produced by marine fungi between 2014 and 2017. We concluded that only two out of 57 molecules were narrow-spectrum Gram^−^ antibiotics [3]. Here, we focus on antimicrobial (antibacterial and antifungal) alkaloids from marine fungi, which more frequently possess a narrow-spectrum Gram^−^ antibiotic activity. A possible explanation as to why this class of molecules holds a higher yield rests in their chemical structure. Small molecules with (primary) amine groups, an amphiphilic nature, rigidity, and low globularity are proven to accumulate more easily inside the cells of Gram^−^ pathogens [8], enabling a more proficient antibacterial activity. Based on their chemical skeleton, alkaloids can be categorized in different classes which are investigated with a different degree of interest. Reviews on pyrrolidine alkaloids [9], marine indole alkaloids [10], and marine diketopiperazines (DKPs) [11,12] were published. This review gives an update on newly discovered alkaloids belonging to those classes, but with a focus on molecules with antimicrobial activity. Alkaloids with antimicrobial activity belonging to the pyrrolizidine, quinazoline, quinoline, and purine classes are also covered. All molecules discussed were derived from marine fungi between 2015 and 2019. Focus is directed toward the isolation strategies and tools, biochemical origin, and structure-activity correlations. The review furthermore highlights the existing knowledge gaps that need to be addressed to elevate alkaloids from marine fungi as promising antimicrobials. 

## 2. New Antimicrobial Alkaloids from Marine Fungi

**Figure 1:**
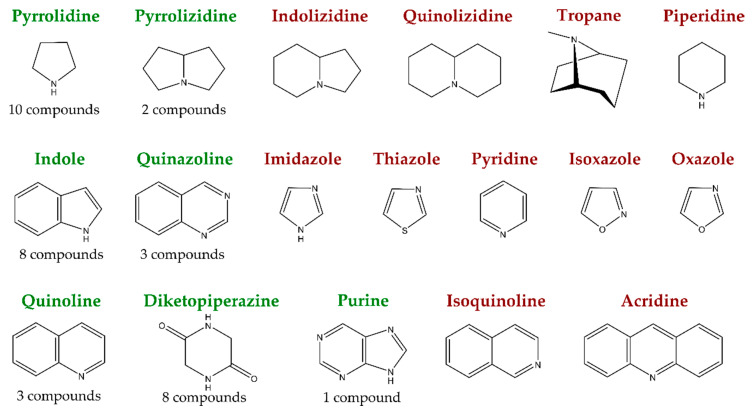
On the left, in green, structural alkaloid groups for which novel compounds with antimicrobial properties are reported since 2015, isolated from marine fungi, alongside with how many compounds in total were found. On the right side, in red, the remaining groups of true alkaloids.

In recent years, many studies concerning alkaloid detection in marine-derived fungi focused on their application potential as cytotoxic agents for the development of innovative cancer therapies, leaving their antimicrobial potential often untapped. In the relatively scarce studies reporting antimicrobial activities for marine-derived fungal alkaloids published between 2015 and today, the following structural groups are represented: pyrrolidine, pyrrolizidine, indole, quinazoline, quinoline, diketopiperazine, and purine alkaloids (Figure 1). These groups are discussed below, and detailed information is given in Table S1 (Supplementary Materials). For the aim of completeness, for all reported compounds, all bioactivities evaluated are listed. 

### 2.1. Pyrrolidines

**Figure 2:**
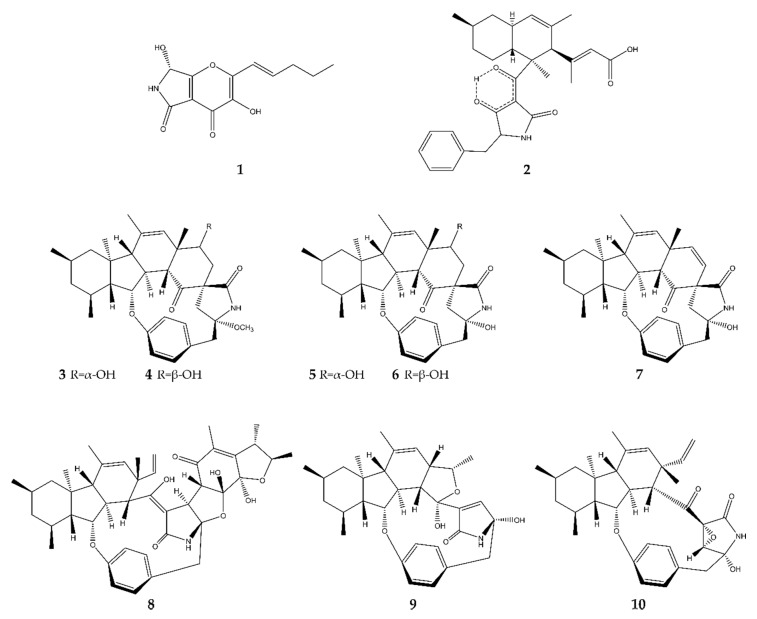
Overview of antimicrobial pyrrolidines in marine fungi discovered between 2015 and the present: pyranonigrin F (**1**), lindgomycin (**2**), pyrrospirone C–F and I (**3**–**7**), penicipyrrodiether A (**8**), penicipyrroether A (**9**), and GKK1032C (**10**).

In total, 10 antimicrobial pyrrolidines were found originating from a variety of marine fungi from around the globe (Figure 2). 

Pyranonigrin F (**1**) was discovered in China by Meng et al. (2015) [13] in the fungus *Penicillium brocae* MA-231 associated with the marine mangrove-derived plant *Avicennia marina* (also known as gray mangrove or white mangrove). This compound is not to be confused with another alkaloid also named pyranonigrin F, which was discovered by Yamamoto et al. in 2015 [14] and where no antimicrobial assay was conducted. After an antimicrobial assessment of **1**, it turned out to possess a potent activity against *S. aureus* (Gram^+^) and the Gram^−^ aqua-bacteria *Vibrio harveyi* and *Vibrio parahemolyticus*, with significantly lower minimum inhibitory concentration (MIC) values than the positive control (chloromycetin). Compound **1** also displayed fungicidal activity against plant pathogens *Alternaria brassicae* and *Colletotrichum gloeosprioides*, again with better MIC values than the positive control (bleomycin). This compound’s cytotoxic activity is yet to be described in the literature [13]. 

Lindgomycin (**2**) was discovered by Wu et al. (2015) [15] in two *Lindgomyces* strains, namely, strains LF327 and KF970, which were isolated from a sponge in the Baltic Sea, Germany, and the Antarctic, respectively. It showed inhibiting activity against the clinically relevant bacteria *Staphylococcus epidermidis*, *S. aureus*, and methicillin-resistant *S. epidermidis* (MRSE). However, the inhibiting activity was two times lower than the positive control chloramphenicol. It is also active against the plant pathogenic bacterium *Xanthomonas campestris*. Compound **2** only targets Gram^+^ bacteria; no inhibition of Gram^−^ bacteria was observed. The human pathogen *Candida albicans* was also repressed, albeit four times less than by the control nystatin. Fungicidal activity was also recorded, against the plant pathogenic fungus *Septoria tritici*. No cytotoxic activity was detected.

Pyrrospirone C–F and I (**3**–**7**) were discovered by Song et al. (2018) [16] in the fungus *Penicillium sp.* ZZ380, which was isolated from a wild crab (*Pachygrapsus crassipes*) living on the seaside rocks of Putuo Mountain (Zhoushan, China). All these compounds inhibited the growth of *E. coli* (Gram^−^) and *S. aureus* (Gram^+^), with **6** being the most potent inhibitor. This means that these compounds possess a broad antibacterial spectrum against both Gram^+^ and Gram^−^ strains. No fungicidal activity was detected. In a sulforhodamine B assay, compounds **3**–**7** showed moderate inhibition of proliferation of glioma U87MG, U251, SHG44, and C6 cells. In this assay, the non-antimicrobial compound pyrrospirone G showed the best activities. In the same fungus, two other antimicrobial alkaloids were discovered by these authors, namely, penicipyrrodiether A [17] in 2018 (**8**) and penicipyrroether A [18] in 2019 (**9**). Both alkaloids have the same antimicrobial spectrum as compounds **3**–**7**. Moreover, both compounds also showed moderate antiglioma activities. Compound **8** inhibited both human gliomas (U87MG, U251, SHG44 cells) and rat gliomas (C6 cells), while **9** was only tested against human glioma U87MG and U251 cells and even showed equivalent inhibitory activity to the positive control doxorubicin (DOX), with a higher selectivity.

GKK1032C (**10**) was discovered by Qi et al. (2019) [19] in the mangrove-associated fungus *Penicillium sp.* CPCC 400,817 in Hainan, China. It displayed activity against both methicillin-resistant and -susceptible *S. aureus*, with activities comparable to the positive control vancomycin. No inhibition of Gram^−^ bacteria was detected.

### 2.2. Pyrrolizidines

**Figure 3:**
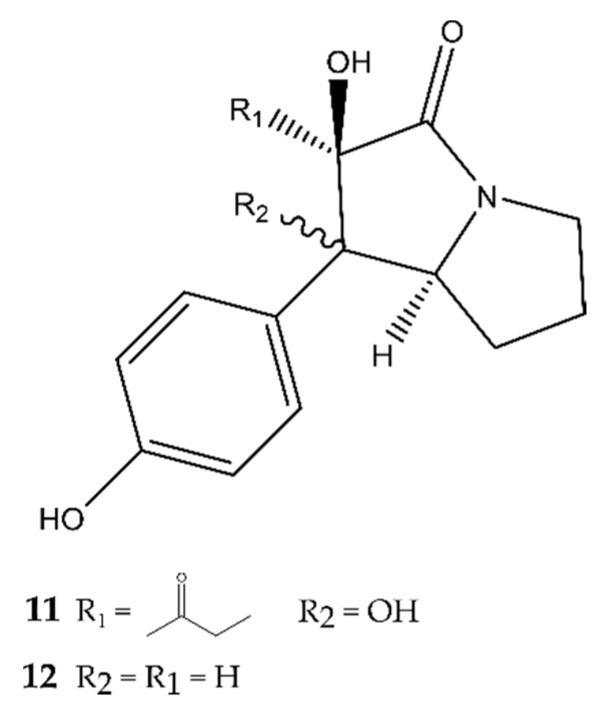
The antimicrobial pyrrolizidines brocapyrrozin A (**11**) and B (**12**).

Only two antimicrobial pyrrolizidines were discovered in the past five years, being brocapyrrozin A and B (**11**–**12**) by Meng et al. (2017) [20] in the fungus *P. brocae* MA-231. This fungus is an endophytic fungus of the marine mangrove plant *A. marina*, discovered in China. In antimicrobial assays, only compound **11** displayed strong inhibiting activity against the clinically relevant bacterium *S. aureus*, even more potent than the positive control chloromycetin. It showed no activity against any Gram^−^ bacteria. Both **11** and **12** possess fungicidal activities against *Fusarium oxysporum*, with **11** being more active than the positive control zeocin and **12** possessing only moderate inhibiting activity. The structure of these compounds is presented in Figure 3. 

### 2.3. Indoles

**Figure 4:**
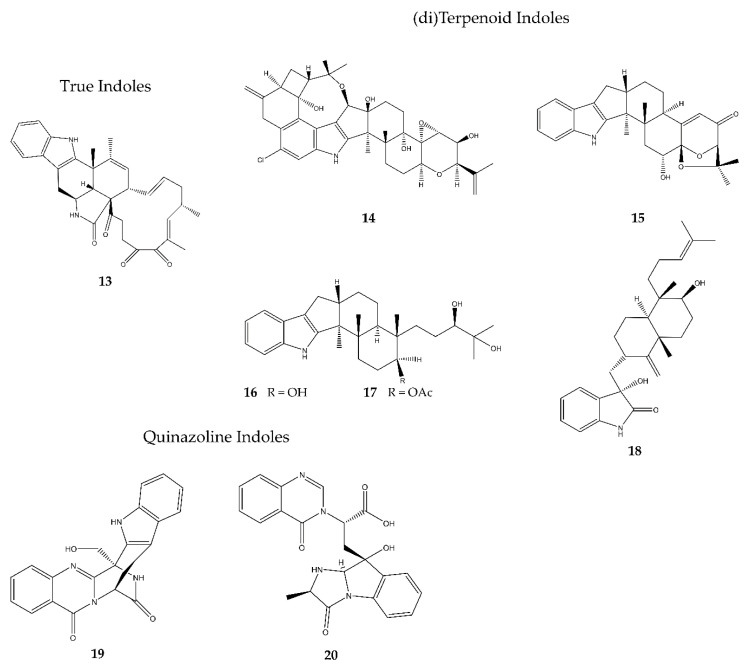
Overview of the different antimicrobial indoles discovered in marine fungi since 2015, divided into three structural classes (true indoles, (di)terpenoid indoles, and quinazoline indoles). In total, eight compounds were discovered, being penochalasin K (**13**), 19-hydroxypenitrem A (**14**), 6-hydroxylpaspalinine (**15**), penijanthine C–D (**16**–**17**), (3*R*,9*S*,12*R*,13*S*,17*S*,18*S*)-2-carbonyl-3-hydroxylemeniveol (**18**), fumigatoside E (**19**), and fumigatoside F (**20**).

A much bigger group of marine-derived antimicrobial alkaloids is represented by the indoles. Eight compounds belonging to this group were discovered over the past five years and had their biological activities studied (Figure 4). Due to a higher structural complexity within this group, it is subdivided into three subgroups: true indoles, (di)terpenoid indoles, and quinazoline indoles.

#### 2.3.1. True Indoles

Penochalasin K (**13**) was discovered by Zhu et al. (2017) [21] in the mangrove-associated fungus *Penicillium chrysogenum* in China. It turned out to be an effective fungicidal compound against *C. gloeosporioides* and *Rhizoctonia solani* with ten-fold and two-fold higher activities than the positive control carbendazim, respectively. Only moderate inhibition of *Colletotrichum musae* and *Penicillium italicum* was observed. It also showed broad-spectrum inhibitory activities against three human tumor cell lines, being a human breast cancer cell line (MDA-MB-435), a human gastric cancer cell line (SGC-7901), and a human lung adenocarcinoma cell line (A549).

#### 2.3.2. (di)Terpenoid Indoles

19-Hydroxypenitrem A (**14**) was discovered by Zhang et al. (2015) [22] in the fungus *Aspergillus nidulans* EN-330, which was derived from the marine red alga *Polysiphonia scopulorum var. villum* found at the Yantai coastline, Northern China. This compound showed moderate antibacterial activity against both Gram^+^ and Gram^−^ species, being *S. aureus*, *E. coli*, *Edwardsiella tarda*, and *Vibrio anguillarum*. Compound **14** also showed potent cytotoxic activity against brine shrimp cells. The chloric atom probably is of high importance for both its antimicrobial and its cytotoxic activity, since unchlorinated similar compounds showed much lower inhibition of both bacteria and brine shrimp cells.

6-Hydroxylpaspalinine (**15**) was discovered by Hu et al. (2017) [23] in the fungus *Penicillium sp.* AS-79, which lives in association with sea-anemones on the Qingdao coastline, China. It showed only weak antibacterial activity against the aquatic pathogen *V. parahemolyticus*, which is a Gram^−^ bacterium. Neither Gram^+^ nor fungicidal activity was detected.

In 2019, two new antimicrobial indoles were discovered by Guo et al. [24], called penijanthine C and D (**16**–**17**). They were detected in *Penicillium janthinellum*, living in the sediments of the Bohai Sea, China. They were only tested for their anti-*Vibrio* activities, important bacterial diseases which cause great losses to mariculture production. Compounds **16** and **17** have inhibiting activities against *Vibrio alginolyticus*, *V. anguillarum*, and *V. parahemolyticus*, which are Gram^−^ bacteria. Of both compounds, **16** showed the most potent activities.

(3*R*,9*S*,12*R*,13*S*,17*S*,18*S*)-2-Carbonyl-3-hydroxylemeniveol (**18**) was discovered by Zhang et al. (2019) [25] in the marine-mud inhabiting *Aspergillus versicolor* ZZ761 in China. Its biological activity comprised the inhibition of both bacterial and fungal sources, being *E. coli* (Gram^−^) and *C. albicans*. In a sulforhodamine B assay, however, it seemed not to inhibit the proliferation of human glioma U87MG and U251 cells.

#### 2.3.3. Quinazoline Indoles

In 2018, Limbadri et al. [26] discovered two new antimicrobial indole alkaloids in the deep-sea sediment-derived fungus *Aspergillus fumigatus* SCSIO41012 in the Indian Ocean and named them fumigatoside E (**19**) and fumigatoside F (**20**). Although they are very similar, they displayed different antimicrobial activities. Compound **19** possesses moderate to strong antibacterial activity against *Acinetobacter baumannii* (Gram^−^), *S. aureus* (Gram^+^), and *K. pneumoniae* (Gram^−^), with MIC values close to the positive control streptomycin, as well as moderate antifungal activity against *F. oxysporum sp. cucumerinu* and strong fungicidal activity against *F. oxysporum sp. momordicae*, with the latter more potent than the positive control nystatin. Compound **20**, on the other hand, showed a much narrower inhibiting spectrum, being only active against the Gram^−^ *A. baumannii* with an MIC value close to the positive control. 

### 2.4. Quinazolines

**Figure 5:**
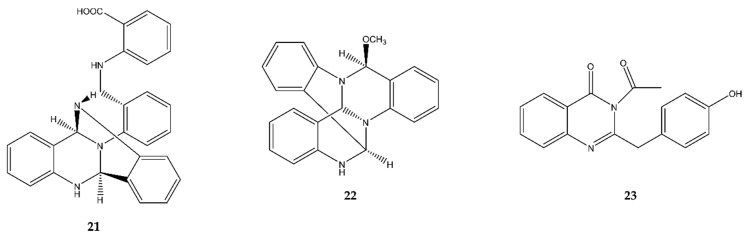
The quinazoline antimicrobial alkaloids oxysporizoline (**21**), thielaviazoline (**22**), and 2-(4-hydroxybenzyl)-4-(3-acetyl)quinazolin-one (**23**).

Three antimicrobial quinazolines were discovered in the past five years in marine fungi (Figure 5).

Oxysporizoline (**21**) was discovered by Nenkep et al. (2016) [27] in the marine fungus *F. oxysporum* in Suncheon Bay, Korea. In an antimicrobial assay, it showed weak antibacterial activity against the Gram^+^, clinically relevant MRSA and multidrug-resistant *S. aureus* (MDRSA). Additionally, it also displayed moderate radical scavenging activity, which was better than that of the positive control, ascorbic acid.

Thielaviazoline (**22**) was discovered by Leutou et al. (2016) [28] in a *Thielavia sp.* strain associated with the marine mudflats of Gomso Bay, Korea. After an antimicrobial assay it showed moderate inhibitory activity against MRSA and MDRSA (Gram^+^). It also showed potent radical scavenging activity with half maximal inhibitory concentration (IC_50_) values higher than the positive control, ascorbic acid.

Liu et al. [29] recently (2019) detected a new antimicrobial quinazoline in *Aspergillus sydowii* SW9, living in China’s seawater. They named the compound 2-(4-hydroxybenzyl)-4-(3-acetyl)quinazolin-one (**23**) and it showed antibacterial inhibition against both Gram^+^ and Gram^−^ bacterial strains. The highest activity was seen against *S. epidermidis*, but *S. aureus*, *E. coli*, and *Streptococcus pneumoniae* were also inhibited by the compound. 

### 2.5. Quinolines

**Figure 6:**
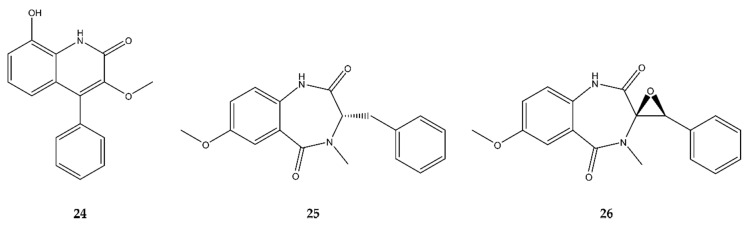
Overview of the different antimicrobial, marine fungal-derived quinoline alkaloids discovered since 2015: 9-hydroxy-3-methoxyviridicatin (**24**), 7-methoxycyclopeptin (**25**), and 7-methoxycyclopenin (**26**).

In 2017, Pan et al. [30] discovered a new antimicrobial quinoline derivative, 9-hydroxy-3-methoxyviridicatin (**24**), alongside its two precursors 7-methoxycyclopeptin (**25**) and 7-methoxycyclopenin (**26**). All compounds were found in the hydrovental crab-associated *Aspergillus versicolor* XZ-4 strain in Taiwan. All three compounds displayed a narrow antibacterial activity, only against the Gram^−^ *E. coli*, with **25** bearing the most potent antibacterial activity of the three, indicating that the viridicatin skeleton is worth exploring further as an antimicrobial compound. The structure of these compounds is presented in Figure 6.

### 2.6. Diketopiperazines

**Figure 7:**
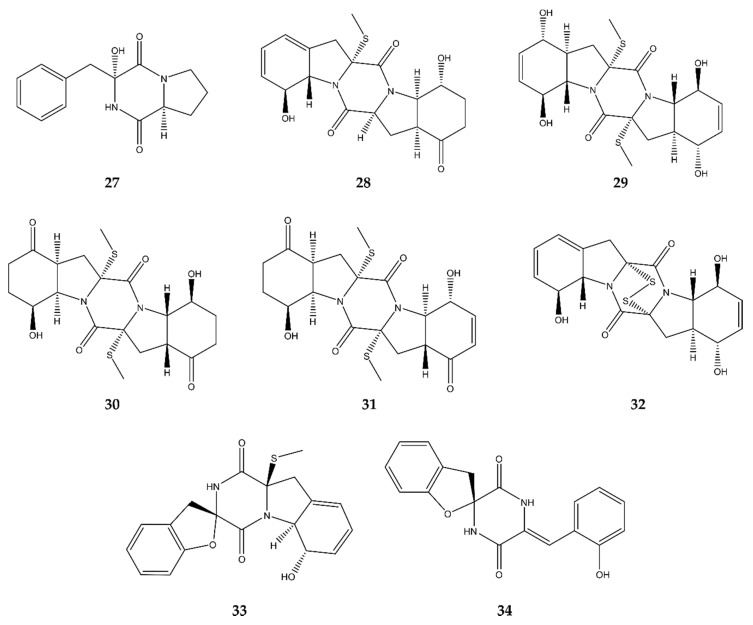
Overview of the different antimicrobial diketopiperazines discovered in marine fungi since 2015: penicillatide B (**27**), penicibrocazine B–E (**28**–**31**), procazine G (**32**), and spirobrocazine A and C (**33**–**34**).

Diketopiperazines (DKPs) could also be seen as simple heterocyclic peptides, which are, in theory, no true alkaloids. However, since diketopiperazines are the outcome of (only) two condensed alpha-amino acids, resulting in a rather simple cyclic system, they still could be seen as alkaloids and, thus, are included in this review. Although their antimicrobial mode of action is often unknown, DKPs have the potential to inhibit bacterial biofilm formation as they can act as quorum-sensing antagonists [31]. Moreover, from the ca. 200 DKPs known in 2014, about 84% were isolated from marine-derived fungi [12]. In total, eight new antimicrobial diketopiperazines were detected in the past five years in marine-derived fungi (Figure 7, compounds **27**–**34**).

Penicillatide B (**27**) was discovered by Youssef et al. (2018) [32] in a non-identified *Penicillium* strain associated with a Red Sea tunicate *Didemnum sp.* This compound showed a very broad-spectrum activity, against both Gram^+^ and Gram^−^ strains, such as *S. aureus* and *V. anguillarum*, respectively. However, its inhibitory activity was more significant against *V. anguillarum* than against *S. aureus*. It also displayed moderate fungicidal activity against species such as *C. albicans*. Moreover, its cytotoxicity and antiproliferative activity against different kinds of human cancer cell lines were tested. It turned out to be significantly active against HCT-116.

In 2015, Meng et al. [33] discovered four new antimicrobial diketopiperazines in the mangrove plant (*A. marina*) associated fungus *P. brocae* MA-231. These compounds were called penicibrocazine B–E (**28**–**31**) and showed different biological activities. Compounds **28** and **30** moderately inhibited the growth of *S. aureus*, with similar activities as the positive control chloromycetin. They also showed more potent fungicidal activity against the plant pathogen *Gaeumannomyces graminis* than the positive control amphotericin B. Thus, **28** and **30** possess a broad-spectrum activity against both Gram^+^ bacteria and fungi. Compound **29** only showed good antimicrobial activity against Gram^+^ bacteria, such as *S. aureus* and *Micrococcus luteus*. In both cases, its MIC value was far below that of the positive control. The last compound (**31**) only possessed fungicidal activity against *G. graminis*. All compounds were also tested for their cytotoxic capabilities against eight tumor cell lines (Du145, HeLa, HepG2, MCF-7, NCI-H460, SGC-7901, SW1990, and U251). None of them showed any potent inhibitory activity.

In 2016, the same research group (Meng et al.) [34] discovered three other diketopiperazines in *P. brocae* MA-231: brocazine G (**32**) and spirobrocazine A and C (**33**–**34**) with different biological activities. Compound **32** showed strong and selective antimicrobial activity, albeit only targeting the Gram^+^ *S. aureus*, while **33** also targeted, in addition to *S. aureus*, Gram^−^ bacteria, such as *E. coli* and *V. harveyi*. Compound **34** on the other hand only displayed antimicrobial activity against Gram^−^ bacteria, such as *E. coli*, *V. harveyi*, and *Aeromonas hydrophila*. Their cytotoxic activities were also measured against both cisplatin-sensitive and -resistant human ovarian cell lines (A2780 and A2780 CisR). Compound **34** showed only moderate activity against the sensitive variant, while **32** showed strong activity against both cell lines, stronger than the positive control cisplatin.

### 2.7. Purines

**Figure 8:**
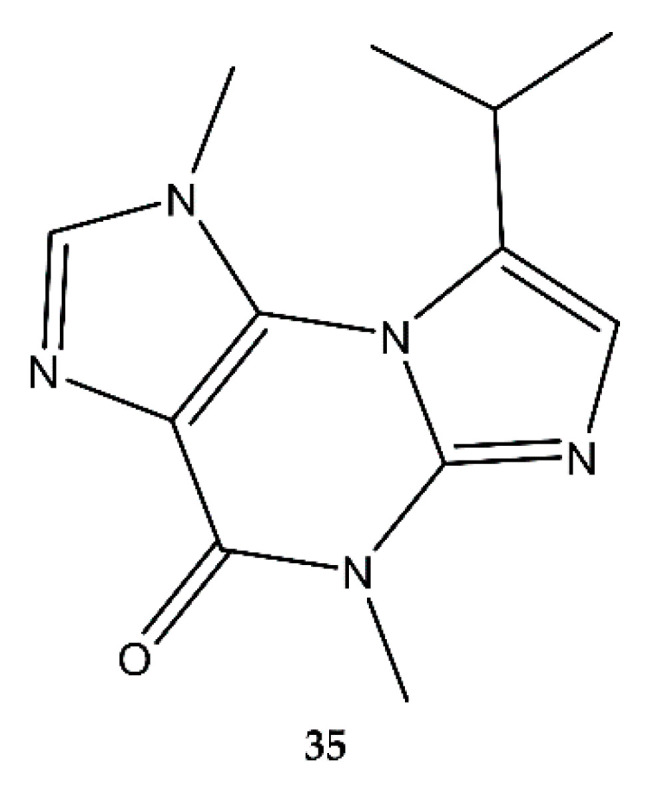
Acremolin B (**35**).

Acremolin B (**35**) was discovered by Tian et al. (2015) [35] in the deep-sea-derived fungus *Aspergillus sp.* SCSIO Ind09F01 (Figure 8). No antimicrobial assay was conducted, only a cytoxicological one. Three years later, Li et al. (2018) [36] detected a purine compound in the Antarctic-derived fungus *A. sydowii* SP-1 and named it acremolin C. It showed weak antibacterial activity exclusively against Gram^+^ bacteria, such as MRSA, MRSE, *S. aureus*, and *S. epidermidis*. No fungicidal activity was detected. In 2019, Banert et al. [37] stated that the compound found by Li et al. (2018) was in fact acremolin B instead of acremolin C, according to their NMR data. This means that acremolin B was found in two different marine fungi and shows antimicrobial activity.

## 3. Natural Product Discovery Strategies for Novel Alkaloids from Marine Fungi

### 3.1. Isolation from Natrural Producers

In the past five years, all antimicrobial alkaloids derived from marine fungi were uncovered using, more or less, the same method (Table S1, Supplementary Materials). The cultured fungus, often stored on solid agar plates such as potato dextrose agar (PDA), rice medium, or Muller Hinton broth agar (MBA), was inoculated in liquid medium in which, more often than not, sea salt was added or seawater was used in the medium preparation, after which it was grown for an excessively long time (14–35 days) in mild growing conditions (25 to 29 °C). The added salt can mostly be seen as a strategy for making the medium more selective, giving the marine fungus an advantage. However, in Liu et al. (2017) [38], it was seen that salt stress could have a complex effect on the secondary metabolism of marine fungi (in this case *Aspergillus glaucus*). They proved the downregulation of terpenoid biosynthesis under salt stress, while the other secondary metabolites remained largely unaffected. It was concluded that, in this case, secondary metabolism was mainly affected by metal ions and the appropriate amounts of certain trace elements. In 2018, Gu et al. [39] discovered a similar effect on the secondary metabolism of *Penicillium citrinum*, as well as a possible activation of silent metabolites. It was found that, after addition of rare earth metals to the culture medium (here, scandium chloride), newly produced metabolites were found in the culture medium, exhibiting better antibacterial activity than those present in the original extract.

In Meng et al. [34], a more advanced strategy was used, described as “one strain, many compounds” (OSMAC). In 2015, this research group found many new alkaloid compounds in extracts of the fungus *P. brocae* MA-231. Pyranonigrin F [13] and penicibrocazines A–E [33] were discovered after the fungus was grown in potato dextrose broth (PDB) medium, prepared by adding naturally sampled seawater. By changing the medium composition to Czapek medium, new compounds could be identified, i.e., spirobrocazins A–C [34] and brocapyrrozins A and B [20]. This could prove to be a viable, easy method to discover new antimicrobial alkaloids in known fungi, by growing them in other production media and analyzing their metabolome. Sometimes, a certain chemical inducer molecule could be added to the medium in order to enhance secondary metabolism (e.g., goadsporin with *Streptomyces* [40]). However, such an inducer compound firstly has to be found for each of these specific microorganisms, which could prove to be time-consuming. 

To increase screening capacity, the isolation Chip (iChip) technique could also be used, in which a chip containing hundreds of holes is covered by a membrane to keep the microorganisms inside. This membrane allows the flow of nutrients, making it possible to maintain the medium as close to the microorganism’s natural environment as possible. Thus far, this technique was only used with bacterial cultures and not fungi.

Co-cultures are another possibility, in which bacterial–fungal interactions are allowed, which are sometimes necessary for the activation of certain secondary metabolites, especially antimicrobial ones. More details on the iChip and co-culture strategies can be found in Lodhi et al. (2018) [41].

Other environmental conditions, such as aeration, temperature, and light, could also be changed to find new alkaloids or to change the spectrum of alkaloids produced by a host.

### 3.2. Isolation from Engineered Strains

Apart from growing conditions, other factors may affect the expression of certain biosynthetic pathways, making their metabolites hard to detect. Several engineering strategies were described in the literature in order to overcome this hurdle. For example, knocking out competitive gene clusters or enhancing the expression of crucial genes (e.g., transcription factors) can allow the discovery of otherwise repressed antimicrobial alkaloids. In 2007, Bergmann et al. [42] discovered two novel alkaloid metabolites, aspyridones A and B, via overexpression of a pathway-specific regulatory gene *adpR*, encoding a transcription factor in *A. nidulans*. This strategy could also prove to be useful in the search for novel antimicrobial alkaloids. However, these strategies are often tedious and costly due to low efficiencies of genetic modification methods. Moreover, for many fungi, such methods are not available, and knowledge on biosynthetic pathways is very scarce, complicating the decision on where to intervene. To bypass this, reconstruction of certain biosynthetic pathways in tractable fungi was developed. Whole-cluster transfer or the expression of large genomic fragments, e.g., from sheared genomic DNA libraries, in more domesticated fungi are also options. The fungal artificial chromosome (FAC)-MS technique was first reported by Clevenger et al. (2017) [43], who screened the genome of several *Aspergillus* species by fractionating and expressing them in the tractable fungus *A. nidulans*, discovering 56 secondary metabolite biosynthesis gene clusters (BGCs). By doing so, 17 new compounds were identified along with their proposed biosynthesis clusters, of which most were silent under previous conditions [43]. Nevertheless, such techniques are arduous and time-consuming, their efficiency remains low. and the hosts apt to use them in are scarce. In Deng et al. (2020) [44], an update on the latest technologies available to identify new metabolites in filamentous fungi was given. The authors proposed the design-construction-evaluation-optimization (DCEO) approach, taking advantage of the current boom in synthetic and system biology. This computer-aided approach offers a variety of methods for the enhanced discovery of silent metabolites in fungi, as well as their biosynthesis through an amalgamation of pathway design/elucidation, pathway construction, pathway evaluation, and pathway optimization. To date, this DCEO approach was optimized for use in filamentous fungi and was already applied in the discovery of organic acids, non-ribosomal peptides, and polyketides. Yet, it could also prove its use in the discovery of antimicrobial alkaloids from filamentous marine fungi [44].

Of classic chemical mutagenesis strategies such as ultraviolet (UV) radiation, formaldehyde, and polychlorinated biphenyls (PCB), there is no mention in the covered timeframe of 2015–2019. However, in future research, these might also prove to be valuable strategies for the discovery of novel antimicrobial alkaloids.

### 3.3. Computational Tools

As discussed in Section 3.1, the discovery of all 35 alkaloid components mentioned in Section 2 still heavily relied on culture-dependent methods. Cultivability of marine fungi, however, remains one of the main bottlenecks for natural product discovery from this rich environment, leaving the huge potential of the so-called “microbial dark matter” untapped [45]. Currently, a shift from traditional screening methods toward bioinformatic-based screening approaches is being observed, resulting in a wide range of computational tools to discover and identify new natural products. Spectacular developments in sequencing technology made it possible to eliminate the classical culturing step and directly mine the genome for the identification of biosynthesis gene clusters (BGCs), using bioinformatic pipelines. To feed these pipelines, data from public repositories (e.g., National Center for Biotechnology Information (NCBI), Kyoto Encyclopedia of Genes and Genomes (KEGG), FungiEnsembl) need to be mined, or supplementary genomic data need to be provided by the user after sequencing the genome of dedicated fungi. Metagenomics approaches could also be considered; however, this could prove to be particularly challenging to obtain fully contiguous BGCs, since it is still very difficult to assemble these vast sequencing reads into contigs per microorganism. BiosyntheticSPAdes is an assembler especially designed for the assembly of BGCs from metagenomic data [46].

For an extensive list of bioinformatic tools suited to discover marine products, the reader is referred to the recent review of Ambrosino et al. [47]. Focusing on those used in the search for new fungal alkaloids from marine environments, antiSMASH is a prominent example of a commonly used secondary metabolite genome mining pipeline. By applying a high-confidence/low-novelty algorithm, known BGCs can be detected with high accuracy. For example, Tang and colleagues used antiSMASH to predict BGCs in the genome of *Penicillium thymicola* IBT 5891, which eventually led to the discovery of the pyranonigrin A biosynthesis gene cluster [48]. Other tools include NaPDoS, which uses the same principle as antiSMASH but is primarily focused at secondary metabolites with polyketide synthases (PKSs) and non-ribosomal peptide synthetases (NRPSs) in their corresponding pathways, and 2ndFind, a web-based secondary metabolite BGC finder. Liu et al. (2015) used the 2ndFind program in their search for the BGC of penitrem A (a member of the fungal indole diterpenes) [49]. Otherwise, genome mining tools based on a low-confidence/high-novelty algorithm might be more desirable if one wishes to detect unknown types of BGCs that remain “silent” under the previous algorithm. ClusterFinder is an example of such a tool. For a more in-depth comparison of the above-mentioned algorithms, the reader is referred to the review of Medema and Fischbach [50]. 

After in silico elucidation of the BGC, confirmation thereof still needs to be carried out. This could be achieved by, for example, enzyme tests, knock-outs, or the transformation of (parts of) the BGC into a tractable host. Strategies on the latter can be found in Section 3.2. An example of a gene knock-out strategy for confirmation of a BGC was published by Yun et al. in 2015. They knocked out the predicted gene responsible for the production of tenuazonic acid (TeA), leading to the abolishment of TeA production and, thus, the confirmation of the gene function [51]. Regardless of which strategy one decides to follow, confirmation of the chemical structure of the produced compound (or absence of it) is crucial. Methods suitable in this regard are discussed in Section 3.4.

### 3.4. Compound Identification

When comparing the analytical protocols used to identify and characterize the 35 antimicrobial alkaloid compounds mentioned in Section 2, it can be concluded that a highly similar approach was used for all. The general outline of this approach comprises separation, extraction, purification, high-resolution electrospray ionization mass spectrometry (HR-ESI-MS), NMR, and confirmation of the structure. Below, each of these steps is briefly discussed.

After a fermentation, with a natural or an engineered host, whether or not in the presence of the initiating compounds mentioned above in Section 3.1, the mycelia are separated from the broth by use of a filter (e.g., cheesecloth filter) or by centrifugation. Afterward, the mycelia are extracted with acetone or methanol (several times), leading to a crude mycelium extract after evaporation of the solvents (and, thus, concentration). The fermentation broth is extracted separately with ethylacetate (EtOAc) (also multiple times) leading to the crude broth extract. A thin-layer chromatography (TLC) or HPLC profile of both crude extracts is often highly similar, and more often than not the recombination of both into one can be done. 

Before being able to analyze and identify the different compounds present in the crude extract, it has to be purified. This extract is initially separated into different fractions using chromatography with a series of elution solvents varying in degree of polarity (e.g., petroleum to methanol or from 70% MeOH in H_2_O to pure MeOH). Examples include ODS (octadecylsilyl groups; reversed-phase C-18), VLC (vacuum liquid chromatography), MPLC (medium-performance liquid chromatography), silica gel flash chromatography, and simple LC (liquid chromatography) or CC (column chromatography). Only in a single case (fumitremorgin C vs. tryprostatin B), an inseparable mixture of two compounds was obtained. In this case, a simple chemical separation method was used; addition of a BOC (*tert*-butyloxycarbonyl) protecting group led to the compounds being separable by use of semi-preparative HPLC. After separation, the BOC group was removed again [52]. After the initial separation, consecutive separation steps are carried out, dividing each fraction into additional subfractions by combination of different kinds of column chromatography (e.g., Lobar LiChroprep RP-18, Sephadex LH-20, ODS) and (semi-)preparative reversed-phase HPLC, until a single compound is obtained. 

When a pure enough compound is obtained from the extract, the identification of its chemical structure and its planar orientation can be obtained. The first step is often an analysis in an MS with a soft ionization source (e.g., HR-ESI or HR-time-of-flight), which can be combined with the degree of saturation, obtained from the ^13^C-NMR spectrum, in order to calculate the chemical molecular formula. The latter can be combined with ^1^H-NMR spectral data in a one- and two-dimensional (1D and 2D) data analysis (e.g., ^1^H–^1^H COSY, HMBC, HSQC, NOESY, distortionless enhancement by polarization transfer (DEPT)) in order to determine the compound’s chemical and planar structure. After obtaining a chemical structure, confirmation of it can be achieved by different means, depending on the purity of the compound. Only highly pure compounds can be subjected to X-ray crystallographic analysis. If the purity of the compound is not high enough, a theoretical ECD (electronic circular dichroism) spectrum can be calculated (e.g., time-dependent density functional theory (TDDFT) model) and compared to its experimental spectrum in order to determine the absolute configuration of the compound.

## 4. Antimicrobial Alkaloids from Marine Fungi and Their Proposed Biochemical Origin 

**Figure 9:**
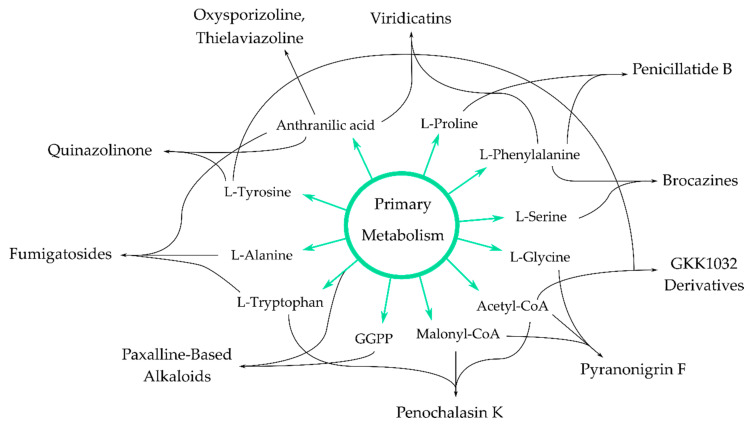
Schematic representation of the proposed biochemical origin of the antimicrobial alkaloids discussed in this review, discovered from 2015 until today.

To date, the biochemical synthesis of compounds discussed throughout this review is not fully elucidated. In some cases, plausible biosynthesis pathways are hypothesized based on the structural classification of these alkaloids, while, for others, biosynthesis gene clusters are proposed and, for some, only speculations are reported. Generally, alkaloids are secondary metabolites biosynthesized from amino acids. Their synthesis is intertwined with the primary metabolism through, e.g., the Krebs cycle and shikimic acid pathway [53]. Recent reviews about the biosynthesis of heterocycles [54], fungal indole alkaloids [55], diterpenoid indole alkaloids [56], and epidithiodioxopiperazines [57] were published. This review identifies the knowledge gaps in the biochemical synthesis routes of newly discovered antimicrobial alkaloids discussed in Section 2 and addresses chemical production strategies if known. The proposed biochemical origin of these novel alkaloids is given in Figure 9, with their biosynthesis gene clusters and those of some closely related compounds in Figure S1 (Supplementary Materials). 

### 4.1. Pyrrolidines

While pyrrolidine alkaloids from plant and animal origin are synthesized from l-glutamate and l-arginine, respectively, via the non-protein amino acid l-ornithine [53], this is not the case for the fungal pyranonigrins. Pyranonigrins possess a pyrano [2,3-c]pyrrole bicyclic skeleton, which is only found in fungal secondary products and mainly in those of *Aspergillus sp.* [13]. In 2015, two independent research groups discovered and named a new pyranonigrin compound “pyranonigrin F”. Meng and colleagues found one with a 1-pentenyl side chain at C-2 (**1**), while Yamamoto and coworkers isolated a spontaneous dimer of pyranonigrin E that contains a cyclobutane through an intermolecular [2+2] cycloaddition [14]. Pyranonigrins can be classified as pyranonigrin A or pyranonigrin E derivatives. The difference between both is the chain length at C-2 and the substitution at C-7. Pyranonigrin A possesses a shorter chain and a hydroxyl group at C-7, while pyranonigrin E is decorated with a longer side chain and an *exo*-methylene group at C-7 [58]. Starting materials for pyranonigrin A are one unit of acetyl-CoA, three units of malonyl-CoA, and one unit of l-glycine [59], whereas pyranonigrin E is biosynthesized from six malonyl-CoA, one acetyl-CoA, and one l-serine unit [58]. The pyranonigrin A biosynthesis gene cluster (BGC) of *P. thymicola* consists of four genes, of which only three are essential for pyranonigrin A production [48]. The polyketide synthase non-ribosomal peptide synthetase (PKS-NRPS) pyrA produces a tetramic acid intermediate, possibly in conjuncture with the adscititious hydrolase pyrD. Subsequently, the flavin-dependent monooxygenase pyrC performs an epoxidation-mediated cyclization resulting in the pyrano [2,3-c]pyrrole skeleton. The cytochrome P450 oxidase pyrB finally carries out a dehydrogenation and a hydroxylation to obtain pyranonigrin S and A, respectively [48,59]. On the other hand, the biochemical synthesis of pyranonigrin E is more complex. The *pyn* cluster of *A. nidulans* is composed of the genes coding for the PKS-NRPS pynA, the flavin adenine dinucleotide (FAD)-dependent oxidoreductase pynB, the *N*-methyltransferase pynC, the cytochrome P450 oxidase pynD, the nicotinamide adenine dinucleotide (NAD)-binding protein pynE, the FAD-containing monooxygenase pynG, the aspartyl protease pynH, the thioesterase pynI, and the putative transcription regulator pynR [14,58]. Similar to pyranonigrin A, a tetramic acid intermediate is synthesized by pynA, which requires pynI for its release. The spontaneous formation of the γ-pyranone precursor pyranonigrin J allows production of pyranonigrin I through the activity of the *N*-methyltransferase pynC. Subsequently, pynG performs an epoxidation-mediated cyclization, the result of which is oxidized by pynD to yield pyranonigrin H. Dehydration of the serine side chain by pynH results in pyranonigrin E with its C-7 *exo*-methylene moiety. The pynE protein is likely to aid pynD in its oxygenase reaction. The reversed oxygenase reaction is catalyzed by the pynB protein. Although the biogenetic origin of pyranonigrin F isolated by Meng and colleagues, with proven antimicrobial activity, is not yet elucidated, the substitutions at positions C-2 and C-7 indicate it can be categorized as a pyranonigrin A derivative.

Contrary to pyranonigrin F, the biochemical origin of the novel pyrrolidine lindgomycin (**2**) is yet to be elucidated [15]. However, during structural analysis, similarities with phomatesin and equisetin analogues, which contain a bicyclic hydrocarbon and a tetramic acid domain, were observed. It was also proven that the original producer strain KF970 produces lindgomycin during the exponential phase and that production is stimulated by a high nitrogen/carbon ratio, low pH, and stable aeration levels. Yet, a deeper understanding of the lindgomycin biosynthesis is still lacking.

A third type of pyrrolidine alkaloids discovered since 2015, the pyrrospirones, contains a 13-membered macroether ring composed of decahydrofluorene, *para*-cyclophane, and pyrrolidinone [18]. The biosynthesis of pyrrospirones C–F and I (**3**–**7**) is assumed to be derived from GKK1032A_1_ or GKK1032A_2_ through oxidation of the vinyl group and electrophilic cyclization of the resulting epoxide [16]. In 2018, penicipyrrodiether A (**8**), a novel adduct of GKK1032A_2_, was discovered in the extracts of *Penicillium sp.* ZZ380, wherein a derivative of phenol A was attached to the pyrrolidinone core through a tetrahydrofuran ring [17]. Song et al. postulated that phenol A is firstly oxidized, after which an intramolecular hemiketal formation takes place to obtain a benzofuran. Tautomerization, subsequent Michael addition of this benzofuran to a dehydro-precursor of GKK1032A_2_, and another intramolecular hemiketal formation then possibly result in penicipyrrodiether A. In addition to penicipyrrodiether A, penicipyrroether A (**9**) was reported in 2019 [18]. Although not an adduct of GKK1032A_2_, it does potentially have a similar biogenetic origin, as the same dehydro-precursor of GKK1032A_2_ as mentioned above could be oxidized to obtain an epoxide intermediate which allows for an intramolecular hemiketal formation to yield penicipyrroether A. Finally, GKK1032C (**10**) was discovered in the extracts of *Penicillium sp.* CPCC 400,817 as a new member of the GKK1032 family, of which biochemical synthesis pathways are still indefinite to date. Through labeling studies, Oikawa elucidated that GKK1032s are produced from l-tyrosine and nine acetate units, as well as l-methionine as a methyl donor [60]; however, more details on the biosynthesis route of these compounds are not known. On the other hand, many chemical syntheses were reported for GKK1032s or their intermediates [61,62,63], as well as for hirsutellones [64,65,66,67,68,69], which are also composed of this unique macroether ether ring. Pyrrospirones and pyrrocidines [70,71] were often neglected since the discovery of these four types of decahydrofluorene-type natural products over 20 years ago. An overview of these studies up until 2013 can be found in the review of Li et al. [72]. Since then, Uchiro and coworkers published an improvement on their GKK1032A_2_ production through an intramolecular nucleophilic aromatic substitution [63]. The C13-hydroxyl group showed reduced reactivity during the cyclization, presumably due to steric hindrance of the remainder of the decahydrofluorene skeleton, resulting in only 15% yield of GKK1032A_2_. By making use of an arenechromium complex for intramolecular nucleophilic aromatic substitution, this cyclization step itself showed a yield of 91% instead of the previously reported 42% [65], improving GKK1032A_2_ yield.

### 4.2. Pyrrolizidines

In 2017, the pyrrolizidines brocapyrrozin A and B (**11**–**12**), which are *p*-hydroxyphenopyrrozin derivatives, were reported [20]. Although phenopyrrozin was first isolated in 1995, nothing is known to date concerning its biochemical origin [73]. However, two chemical syntheses for *p*-hydroxyphenopyrrozin were reported. The first starts from l-proline and requires 11 steps to obtain a 5.8% yield of *p*-hydroxyphenopyrrozin [74], while the second makes use of a three-step condensation cyclization of α-oxoesters and cyclic imines, resulting in an overall yield of 55% [75].

### 4.3. Indoles

In addition to pyrrolidine and pyrrolizidine alkaloids, eight new antimicrobial indoles were discovered since 2015: five terpenoid indoles, two quinazoline indoles, and one true indole, penochalasin K.

The biosynthesis pathway in *P. chrysogenum* for penochalasin K (**13**), a chaetoglobosin, was hypothesized to be branched from the one of chaetoglobosin C through an additional epoxide ring opening, dehydration elimination, and electrophilic substitution [21]. Evidence to bolster this hypothesis was delivered by a three-step chemical conversion of chaetoglobosin C into penochalasin K: (i) sodium carbonate at room temperature for half an hour, (ii) dimethylsulfate at 40 °C for 2 h, and (iii) pH alteration through ammonia water. Replacing the dimethylsulfate solution with sulfuric acid was also feasible, although a decrease in penochalasin K yield from 36.9% to 8.3% was observed. The same chemical conversion was used in the alteration of chaetoglobosin A into chaetoglobosin I, a structural similar compound to penochalasin K. A series of (de)hydrations, (de)protonations, and rearrangements to explain this conversion was given. The biogenetic pathway for chaetoglobosins starts from acetyl-CoA, malonyl-CoA, and l-tryptophan to establish prochaetoglobosins and other intermediates of this bioactive class of compounds [76,77,78]. The BGC for chaetoglobosin A was identified in two terrestrial endophytic fungi: *Penicillium expansum* and *Chaetomium globosum*. The former contains the *che* BGC which consists of the genes coding for the PKS-NRPS CheA, the enoyl reductase CheB, the regulators CheC and CheF, the cytochrome P450 monooxygenases CheD and CheG, and the FAD-dependent monooxygenase CheE (Genbank AM779763.1) [79]. Similarly, the latter contains a BGC encoding a PKS-NRPS (CHGG_01239), an enoyl reductase (CHGG_01240), two cytochrome P450 monooxygenases (CHGG_01242-1 and CHGG_01243), and an FAD-dependent monooxygenase (CHGG_01242-2) [80]. Additionally, a transcription factor (CHGG_01237), a transposase (CHGG_01238), and two genes with unknown function were reported. Although the genomes of *P. expansum*, *P. chrysogenum*, and *C. globosum* were sequenced, the BGC for chaetoglobosin C still remains elusive.

Diterpenoid indoles are typically synthesized from indole-3-glycerol phosphate, which is a precursor of l-tryptophan and geranylgeranyl diphosphate [22]. Penitrem A is a tremorgenic diterpenoid indole composed of a paxilline core with a tricyclic penitrem skeleton. In 2015, two BGCs for penitrem A production were discovered in *Penicillium simplicissimum* containing 20 genes in total [49]. The first cluster (Genbank LC027936.1) contains the genes *ptmGTVHINDAQMBCPSU* while the second entails *ptmOJKEL* (Genbank LC027937.1). Production starts from indole-3-glycerole phosphate and farnesyl diphosphate, the precursor of geranylgeranyl diphosphate, to yield the paxilline skeleton, catalyzed by the enzymes ptmGCMBPQ. During a second stage, paxilline is conversed through cyclization to the bicycle[3.2.0]heptane of the penitrem core (ptmHVIOE). Subsequently, epoxidation, hydroxylation, and chlorination result in penitrem A formation (ptmKULJ). How penitrem A is converted into 19-hydroxypenitrem A (**14**), the new antimicrobial alkaloid mentioned in Section 2, is not reported to date.

Paspalinine has the same biochemical origin as paxilline. From the penultimate reaction in the synthesis of paxilline, which generates 13-desoxypaxilline, only two additional steps are required to form paspalinine [56]. Both steps are catalyzed by the cytochrome P450 monooxygenase atmQ, present in the BGCs for aflatrem, a diterpenoid indole for which paspalinine is a precursor. Similar to the penitrem A BGCs, the aflatrem genes are also divided on two genomic locations in the *Aspergillus flavus* and *A. oryzae* genomes (Genbank: AY559849.2 and AM921700.1) [81]. Conversion of paspalinine to 6-hydroxylpaspalinine (**15**) is not known.

Other derivatives of paxilline analogues are the panijanthines. Since the discovery of panijanthine A in 2009 [82], only the structural elucidation of panijanthines B–D was reported [24,83]. Consequently, no information is available on the biochemical origin of panijanthines, apart from the fact that they are structurally similar to paxilline and, thus, could have a similar biochemical origin [82]. Furthermore, the biosynthesis of (3*R*,9*S*,12*R*,13*S*,17*S*,18*S*)-2-carbonyl-3-hydroxylemeniveol (**18**) is not yet investigated so far. However, paspaline (and, thus, paxilline) was proposed as a precursor of the structurally related indole emeniveol, which was first isolated in 1992 [84]. The total chemical synthesis of emeniveol starting from a (+)-Wieland–Miescher ketone was reported in 1993 [85]. Additional information concerning the structural diversity and biological activity of tremorgenic diterpenoid indoles like the ones discussed above was described by Reddy et al., while further efforts toward their chemical synthesis were reported by Zou and Smith [86].

Fumigatosides, quinazoline indoles, were originally discovered as glycosylated fumiquinazolines and were implied as their precursors [87]. These fumiquinazolines consist of a quinazolinone core fused to a piperazine or spiro ring. Their biochemical origin entails anthranilic acid, l-tryptophan, and l-alanine, and their biosynthesis was recently reviewed by Resende and colleagues [88]. The BGC of fumiquinazolines in *A. fumigatus* contains, among others, a transporter (Af12040), a monomodular NRPS (Af12050), an FAD-dependent monooxygenase (Af12060), an FAD-dependent oxidoreductase (Af12070), and a trimodular NRPS (Af12080) [89]. In *Penicillium aethiopicum*, the *tqa* BGC of tryptoquialanine shows high similarities with the aforementioned enzymes for fumiquinazoline production in *A. fumigatus* [90]. While fumigatoside A was too instable for structure elucidation, fumigatosides B–D showed comparable features to fumiquinazolines [87], while fumigatosides E and F (**19**–**20**) are not glycosylated [26]. Nevertheless, these two novel tripeptide alkaloids were co-discovered with known fumiquinazolines.

### 4.4. Quinazolines

Of the 35 novel alkaloids discussed in this review, three belong to the quinazoline alkaloids. Oxysporizoline (**21**) and thielaviazoline (**22**) are respectively a *tris*-anhydrotetramer and a trimer of anthranilic acid, for which no biochemical information is available [27,28]. On the other hand, self-condensation of anthranilic acid and chemical synthesis of polycyclic quinazoline derivatives like oxysporizoline complexed with metals were reported, and the latter implied several interesting bioactivities [91]. Another novel quinazoline alkaloid is 2-(4-hydroxybenzyl)-4-(3-acetyl)quinazolinone (**23**), which only differs from the co-discovered 2-(4-hydroxybenzoyl)-4(3H)-quinazolinone through an *N*-acetyl moiety [29]. The latter was discovered in 2011 [92] and its proposed biogenetic origin starts with l-tyrosine [93]. l-Tyrosine might be transferred to 4-hydroxybenzeneacetamide, which in turn performs a nucleophilic addition with anthraniloyl-CoA to generate 2-(4-hydroxybenzoyl)-4(3*H*)-quinazolinone.

### 4.5. Quinolines

**Figure 10:**
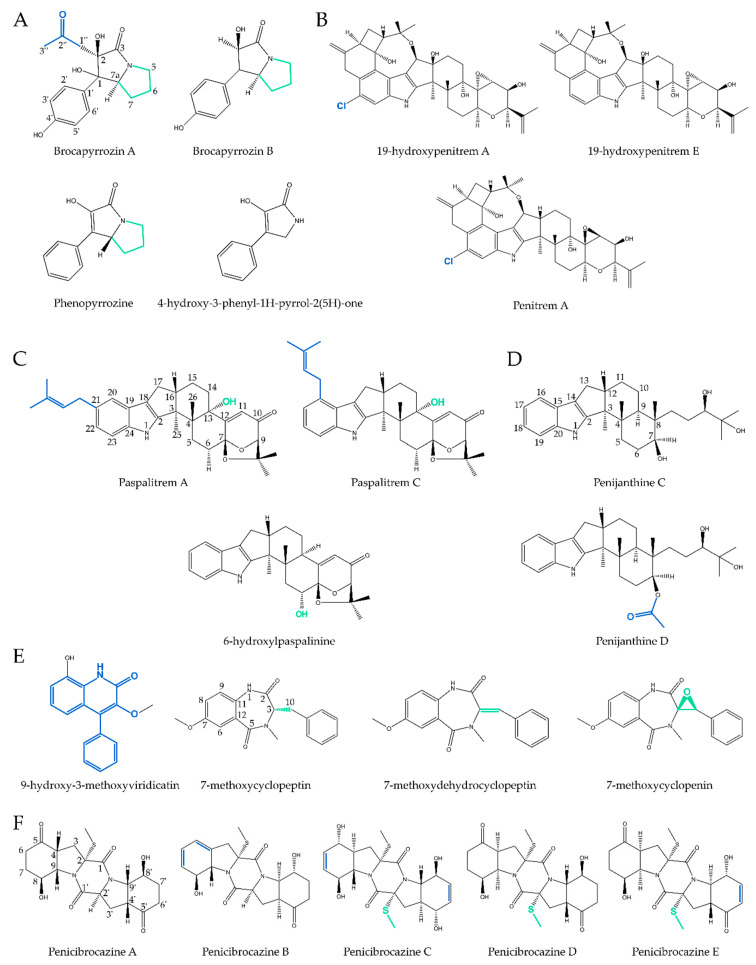
Structure–activity analysis of newly discovered antimicrobial alkaloids discussed in this review. (**A**) Structural comparison of brocapyrrozin A and B, phenopyrrozin, and 4-hydroxy-3-phenyl-1*H*-pyrrol-2(5*H*)-one. The acetonitrile group on C-2 (blue) and the absence of the pyrrole group (green) probably increase the fungicidal activity. (**B**) Structural comparison of penitrem A and its derivatives, 19-hydroxypenitrem A and 19-hydroxypenitrem E. The presence of a chlorine atom (blue) most likely elevates antibacterial activity. (**C**) Structural comparison of paspalitrem A, paspalitrem C, and 6-hydroxylpaspalinine. The differences between the structures are highlighted in blue and green. (**D**) Structural overview of penijanthines C and D. The acetoxy group at C-7 (blue) of penijanthine D probably lowers anti-*Vibrio* activity. (**E**) Structural overview of 9-hydroxy-3-methoxyviridicatin and the cyclopeptin derivatives. The viridicatin (blue) structure is proven to be a promising antibacterial compound core, worth looking further into, while the cyclopeptin antimicrobial activity could be dependent on the chirality of its C-3 atom (green). (**F**) Structural comparison of penicibrocazines A–E. The lack of double bonds (blue) and/or *S*-methyl group at C-2′ (green) might explain the absence of antimicrobial activity of penicibrocazine A compared to penicibrocazines B–E.

In addition to quinazoline alkaloids, quinolines are also synthesized from anthranilic acid. Although quinolines were already discovered in the 1950s, the BGCs of viridicatin and its derivatives were only reported recently [94,95]. The first step toward viridicatin synthesis is a condensation of anthranilic acid and either l-phenylalanine or *O*-methyl-l-tyrosine, catalyzed by an NRPS. The latter results in (−)-4′-methoxycyclopeptin while cyclopeptin is obtained in the case of a condensation with l-phenylalanine [96]. These 6,7-bicyclic cyclopeptins are subsequently converted to respectively (−)-4′-methoxycyclopenin or (−)-cyclopeptin by a dioxygenase. This dioxygenase is very unique as it catalyzes an oxidation and an epoxidation reaction, which are typically performed by monooxygenases [94]. The oxidation reaction is a desaturation which results in a double bond that is successively used in the epoxidation. Conversion of (−)-4′-methoxycyclopenin to 4′-methoxyviridicatin finally occurs spontaneously, while synthesis of viridicatin from (−)-cyclopeptin requires the action of a cyclopenase [96]. 4′-Methoxyviridicatin is a 6,6-quinolone scaffold used in fungi such as *A. nidulans* and *Penicillium sp.* to synthesize other bioactive secondary metabolites such as aspoquinolones [97], peniprequinolones [98], penigequinolones [99], and yaequinolones [100]. The biochemical pathways, together with the enzymes responsible for the synthesis of aspoquinolones and penigequinolones, were documented by Kishimoto et al. The genes encoded in the BGCs are given in Figure 10; however, only the *pen* BGC is accessible on Genbank (KX528209.1). The newly discovered 9-hydroxy-3-methoxyviridicatin (**24**) and its precursors 7-methoxycyclopeptin (**25**) and 7-methoxycyclopenin (**26**) are synthesized from *O*-methoxy-anthranilic acid and l-phenylalanine [30], which presumably entails a biochemical pathway resembling the above-mentioned one for (4′-methoxy)viridicatin production.

In addition to the biosynthetic production of viridicatin and derivatives, chemical conversion is also feasible. Drawbacks are mostly the limited availability of starting material or transition metal and the cumbersome derivatization of functional groups. An overview can be found in the manuscripts of Kobayashi et al. [101] and Tangella et al. [102]. Both manuscripts tackle these drawbacks and report a one-pot synthesis from readily available compounds in a metal-free fashion and with a broad substrate scope. The former makes use of a Knoevenagel condensation and epoxidation to obtain viridicatin derivatives from cyanoacetanilides and benzaldehyde substrates in three steps: (i) condensation with piperidine as catalyst in dimethylformamide at room temperature for 48 h, (ii) epoxidation with *tert*-butyl hydroperoxide and potassium fluoride at room temperature for 24 h, and (iii) cyclization of the epoxide with sulfuric acid [101]. Twelve viridicatin derivatives with a yield of 38–82% were obtained from six cyanoacetanilides and six aldehydes. Ice-cold water was used to precipitate the viridicatin compounds. The latter reports the synthesis of 36 viridicatin alkaloids with a yield up to 96% through a regioselective ring-expansion of isatins. The conversion of an aldehyde and a 5,6-bicyclic isatin indole to the 6,6-bicyclic viridicatin was catalyzed by *p*-toluene sulfonylhydrazide, employing potassium carbonate and ethanol at 80 °C for 8 h. Isatins can be obtained through different chemical strategies, which were discussed by Chahal et al. [103].

### 4.6. Diketopiperazines

Diketopiperazines (DKPs) are biosynthesized as a condensation of two α-amino acids [104]. These compounds occur alone, and then represent the simplest form of peptides, or they are incorporated in larger skeletons. For example, penicillatide B (**27**), the new antimicrobial compound discussed in Section 2, and penicillatide A are standalone DKPs. The former is a condensation of l-proline and l-phenylalanine, while the latter is a condensation of *N*-formylleucine and 2-oxopyrrolidine [32]. Due to their small size and the advance of solid-phase peptide synthesis, DKPs are commonly applied in combinatorial chemistry in the search for novel bioactive compounds. The synthesis of DKPs was previously reviewed thoroughly and is not discussed here [104].

Most of the other diketopiperazines discussed in this review are epithiodioxopiperazines (ETPs), which possess a disulfide bridge or a polysulfide dioxopiperazine ring, and they are unique to fungi [57,105]. Brocazines are bisthiodiketopiperazine derivatives with a disulfide bridge in their 6,5,6,5,6-pentacyclic structure, resembling epicorazine A, which was co-discovered in the extract of *P. brocae* [34,106]. Penicibrocazines [20,33] and spirobrocazines [34] are similar compounds but without a disulfide bridge and, in the case of the latter, containing a rare spirocyclic center. Efforts to chemically synthesize brocazin F and G (**32**) were described by Hulangamuwa [107]. On the other hand, to date, no BGCs are reported for these brocazines or related compounds, including epicorazines. However, the biochemical origin of epicorazine A is found in the condensation of l-serine and l-phenylalanine by a dioxopiperazine synthase [108]. Subsequent sulfurization and multiple oxidations generate an epoxide intermediate, which is required for the apoaraotin A ring, a precursor of epicorazine-type ETPs [105]. The sulfur atoms furthermore originate from l-methionine, l-cysteine, or sodium sulfate, although the manner is yet to be elucidated.

Gene clusters were reported for the closely related ETPs gliotoxin, aranotin, and sirodesmin: *gli*, *ata*, and *sir*, respectively [108,109]. With the exception of sirodesmin, these ETPs are also synthesized from l-serine and l-phenylalanine. Furthermore, methylation patterns of gliotoxin revealed the importance of methyltransferases for ETPs, opening the door for structure–activity speculations of brocazines and related compounds. Methylation by the *S*-adenosyl-methionine (SAM)-dependent *N*-methyltransferase of the *gli* cluster increased the potency and stability of gliotoxin, whereas the SAM-dependent *S*-methyltransferase irreversibly inactivated gliotoxin [110].

### 4.7. Purines

Finally, since 2015, only one purine-derived antimicrobial alkaloid from marine fungi was discovered: acremolin B (**35**) [35]. As accustomed with acremolins [111,112], structure elucidation was not straightforward and resulted in the postulation of acremolin C [36], which was later reoriented to the chemical structure of acremolin B [37]. Although nothing is known about the biosynthetic origin of acremolins, a five-step metal-free chemical synthesis of acremolin starting from guanosine was reported [113]. 1-Methylguanine was obtained through selective methylation and subsequent hydrolysis. The third step entailed the protection of the nitrogen atom at position 7 with *p*-methoxybenzyl (PMB), resulting in a mixture of PMB protection at the nitrogen atom at position 7 or position 9. Through fractional crystallization with ethanol, the former was isolated. Condensation of the PMB-protected 1-methylguanine with 1-bromo-3-methyl-2-butanone resulted in PMB-protected acremolin. Finally, deprotection with TFA yielded acremolin.

## 5. Structure–Activity Analysis

In what follows, a structure–activity-based analysis of antimicrobial alkaloids is reviewed. This could prove to be useful information when designing new, promising antibiotics via derivatization of naturally occurring compounds. For the sake of this review, this analysis is restricted to the compounds reported in Section 2.

Brocapyrrozin A and B (**11**–**12**), as mentioned before, are both active against *F. oxysporum*, with **11** being more potent than **12**. This indicates that the acetonitrile group on C-2 (blue in Figure 10A) of the pyrrolizidine core probably increases the inhibiting activity of brocapyrrozin A against fungi, more specifically *F. oxysporum*. In the same article, it was mentioned that the presence of the pyrrole unit (green in Figure 10A) is likely to decrease the inhibiting activity against *F. oxysporum*. This was hypothesized when comparing phenopyrrozine with 4-hydroxy-3-phenyl-1*H*-pyrrol-2(5H)-one [20]. Perhaps this pyrrole unit also inhibits the activity of brocapyrrozin A and B; thus, removing it could raise the fungicidal activity of brocapyrrozin A even further.

19-Hydroxypenitrem A (**14**), along with 19-hydroxypenitrem E and penitrem A, showed moderate antibacterial activities against both human- and aqua-related pathogens. In Zhang et al. (2015), it was seen that the chlorinated (blue in Figure 10B) compounds, being 19-hydroxypenitrem A and penitrem A, demonstrated better antibacterial activities than the non-chlorinated one, 19-hydroxypenitrem E. This suggests that the chlorine atom is favorable for the antibacterial activity of penitrems and their derivatives [22].

In an antimicrobial assay, 6-hydroxylpaspalinine (**15**) showed only antibacterial activity against *V. parahaemolyticus*, while paspalitrem C exhibited a much more broad-spectrum activity against *V. parahaemolyticus*, but also against *P. aeruginosa*, *E. coli*, and *V. alginolyticus*. When comparing the structure of both paspalinine derivatives (in Figure 10C), it can be seen that they are highly similar and only differ in two places; paspalitrem C possesses a prenyl group at C-20 (blue) and a hydroxyl group at C-13 (green), while 6-hydroxylpaspalinine has a hydroxyl group at C-6. The difference in activity between paspalitrem A and paspalitrem C is even bigger, while their structures are more similar. Apparently, the prenyl group (blue) has to be situated on C-20, for, in paspalitrem, A it is positioned on C-21 and this compound demonstrated no antibacterial activity in the assay [23].

Penijanthines C and D (**16**–**17**) were only tested for their anti-*Vibrio* activity, to assess their potential as vibriosis moderators in marine cultures. It was seen that penijanthine C displayed a higher antibacterial activity against these species, than did D, suggesting that the acetoxy group (blue in Figure 10D), situated at C-7, may be an inhibitor of anti-*Vibrio* activity. After a literature survey, it was concluded that the indole terpenoid derivatives are an interesting group for the search of novel anti-*Vibrio* drugs and need to be looked further into. It was found in other reports that 6-hydroxylpaspalinine, paspalitrem C, and so on also possess anti-*Vibrio* properties, while also being indole terpenoids [24].

In Pan et al., it was seen that 9-hydroxy-3-methoxyviridicatin (**24**) and 3,6-*O*-dimethylviridicatin, both viridicatin (blue core structure in Figure 10E) derivatives, exhibited good antibacterial properties, indicating that the viridicatin skeleton could prove to be worth looking further into. When comparing the antimicrobial activities of three cyclopeptine derivatives, it was concluded that 7-methoxydehydrocyclopeptin showed no activity, while 7-methoxycyclopeptin (**25**) and 7-methoxycyclopenin (**26**) both showed moderate antibacterial activity. The biggest difference between these three molecules being the double bond between C-3 and C-10 (green in Figure 10E in 7-methoxydehydrocyclopeptin suggests that the chirality of C-3 is of importance for the antimicrobial activity of cyclopeptin derivatives [30].

Penicibrocazines A–E were discovered by Meng et al. in 2015 and have varying activity against microorganisms [33]. When comparing A and B (**28**), A has no antimicrobial activity, while B does, indicating that the presence of double bonds (blue in Figure 10F) at C-4 and C-6 or the lack of a keto group at C-5 increases potency. The structure of penicibrocazines C–E (**29**–**31**) is different compared to A and B, as they have an additional *S*-methyl group at C-2′ (green). As this *S*-methyl group is the only difference between penicibrocazine A and D, the presence of the keto group at C-5 does not appear to influence antimicrobial activity. Penicibrocazines C and E also possess double bonds at C-6 and/or C-6′, potentially increasing antimicrobial activity, as might be the case for penicibrocazine B. Despite not being able to unambiguously indicate the importance of double bonds or the *S*-methyl group, both features might play a pivotal part in future structure–activity studies of penicibrocazine alkaloids.

All above-mentioned structure–activity relationships were detected by comparing antimicrobial activities of different structures, making conclusions on sight. However, there also exist in silico tools for the prediction of structure-based activities, called molecular docking tools, that allow for enhanced studies through inclusion of physicochemical properties and three-dimensional (3D) visualization. Examples thereof are UCSF’s DOCK, FlexX4, rDOCK, SwissDock, and many more, which were discussed by Ambrosino and colleagues [47].

## 6. Conclusions

Between 2015 and 2019, 35 novel antimicrobial alkaloids from marine fungi were reported. From these, seven were narrow-spectrum Gram^+^ antibiotics, whereas eight were solely active against Gram^−^ bacteria, and three acted as pure antifungal compounds. Moreover, 10 alkaloids showed a broad-spectrum antibacterial activity, while three were broad-spectrum against Gram^+^, Gram^−^, and fungal microorganisms. As Gram^−^ pathogens are currently the most exigent threat, the high prevalence of narrow-spectrum Gram^−^ antibiotics in marine fungi-derived alkaloids proves that the marine environment should be searched further in the battle against multidrug resistance. Currently, marine fungi are rather undermined pertaining their antimicrobial secondary metabolites, as the main focus seems to be on cytotoxicity studies for cancer and tumor research. Nevertheless, the marine environment has many natural products to offer with a plethora of applications, such as cytotoxic, antioxidant, antimicrobial, antiviral, and anti-fouling compounds, as well as anti-acetylcholinesterases and plant growth stimulators.

The search for antimicrobial compounds in marine environments, specifically produced by marine fungi, should, in several aspects, broaden possibilities. For example, most of the alkaloids discussed throughout this review were isolated from southeast Asia, a geographical bias that needs to be solved. Another myopic aspect of current studies is the natural product discovery strategy. During the past five years, all antimicrobial alkaloids derived from marine fungi were uncovered using, more or less, the same method. A simple change of complex growth medium is often the only treaded path, while other strategies such as the use of inducer compounds proved useful as well. In this review, we propose several other strategies promising in the search for antimicrobial compounds from marine fungi, such as in situ production at the native environment, co-culture techniques, adapting environmental conditions, the use of engineered strains, and a plethora of possibilities emerging from recently developed bioinformatics-based pipelines. Several of these alternatives also allow skipping the 30-day incubation step required for the enrichment of secondary metabolites in the currently preferred methods. Finally, extraction and analytical methods are very similar to each other, introducing another bias in antimicrobial alkaloid discovery in marine fungi.

In addition to novel methods to find them and a deeper characterization of their activity, novel antimicrobial alkaloids derived from marine fungi would also benefit from a more thorough search for their biochemical origin, as this would be a start toward their biobased production. Current reports focus on structure elucidation and little on the biogenetic pathways required for their synthesis. This review, therefore, indicates the gaps between newly discovered alkaloids and known biochemistry. Moreover, an overview of biosynthesis gene clusters of (related) alkaloids is given. To fill these gaps, chemical synthesis strategies or the prevalence of certain genes in other clusters could be used. In case of the latter, it is clear that alkaloid synthesis of marine fungi might rely heavily on flavin-dependent and cytochrome P450 monooxygenases, as well as on polyketide synthases and non-ribosomal peptide synthetases. The chemical conversion of guanosine into acremolin could, e.g., lay the foundation for the elucidation of the biochemical pathways used in its production. Moreover, chemical synthesis strategies and corresponding substrate specificity studies are complementary with natural product discovery as new derivatives with altered efficacy and/or activity from discovered natural products can be investigated.

Finally, a deeper understanding of the correlation between chemical structure and activity is essential. From this review, it is clear that the use of transferases such as the SAM-dependent *N*-methyl or *S*-methyltransferases in the gliotoxin cluster, as well as chemical synthesis strategies that allow for a broad substrate specificity, could prove vital in this regard and elevate antimicrobial alkaloids from marine fungi to the next level.

## Supplementary Materials

The following are available online at https://www.mdpi.com/2079-6382/9/6/340/s1: Table S1. Overview of newly discovered antimicrobial alkaloids from marine fungi published between 2015–2019. Figure S1. Overview of known biosynthesis gene clusters of new antimicrobial alkaloids discussed in this review, and of closely related compounds.
