# Supplementary material for: Alkaloids from Marine Fungi: Promising Antimicrobials
Source: Antibiotics (Basel). 2020 Jun 18;9(6):340. doi: 10.3390/antibiotics9060340 (PMC7345139; doi:10.3390/antibiotics9060340)
Supplement: Supplementary file 1 [file antibiotics-09-00340-s001.zip › Supplementary Figure S1_Biosynthesis gene clusters.docx]

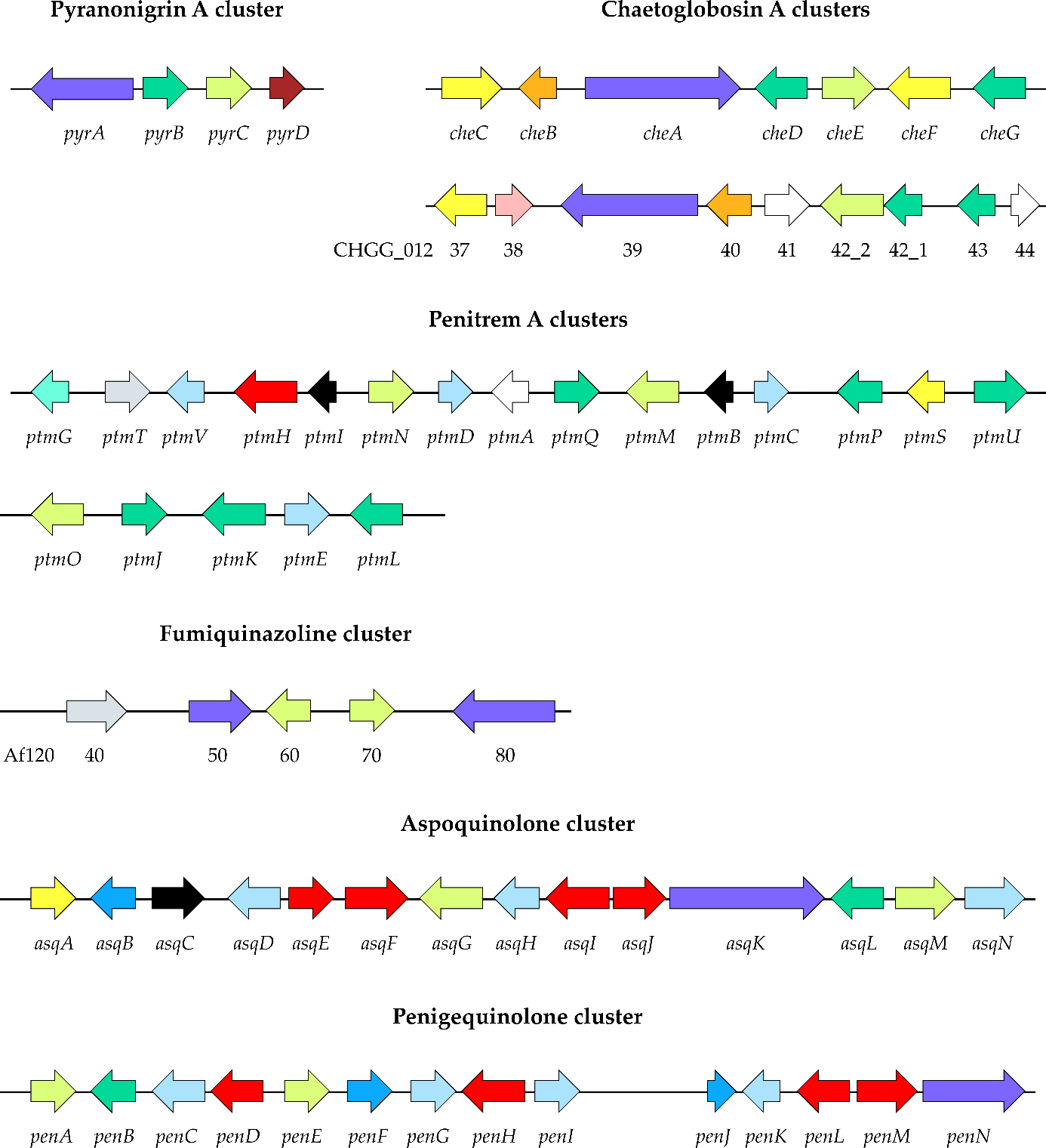


**Supplementary Figure S1.** Overview of known biosynthesis gene clusters of new antimicrobial alkaloids discussed in §2 and of some closely related compounds. In case of the aspoquinolone cluster, *asqO* and *asqP* are not visualized as they were only recently discovered without mention of their relative position in the cluster. Purple: polyketide synthase or nonribosomal peptide synthetase; olive green: cytochrome P450 monooxygenase; light green: flavin-dependent monooxygenase; cyan: geranygeranyl pyrophosphate synthase; dark blue: hydrolase; light blue: transferase; crimson red: thioesterase; red: oxidoreductase; pink: transposase; orange: enoyl reductase; yellow: regulator; black: cyclase; grey: transporter; and white: hypothetical protein or unknown function.
