# Supplementary material for: Alkaloids from Marine Fungi: Promising Antimicrobials
Source: Antibiotics (Basel). 2020 Jun 18;9(6):340. doi: 10.3390/antibiotics9060340 (PMC7345139; doi:10.3390/antibiotics9060340)
Supplement: Supplementary file 1 [file antibiotics-09-00340-s001.zip › Supplementary Table S1_Novel antimicrobial alkaloids.docx]

Supplementary Table S1: Overview of newly discovered antimicrobial alkaloids from marine fungi published between 2015−2019. The coverage indicates which microorganisms were susceptible to the compounds.

|  | Compound | Alkaloid type | Origin | Producer | Discovery method | Coverage | Spectrum | Gene cluster (Genbank n°) | Reference |
| --- | --- | --- | --- | --- | --- | --- | --- | --- | --- |
| 1. | Pyranonigrin F | Pyrrolidine | Mangrove plant, China | *Penicillium brocae MA-231* | Medium & inc. | *ABr, CG, SA, VH, VP* | F, Gram+ & Gram^−^ | *pyr* | Meng *et al*., 2015 |
| 2. | Lindgomycin | Pyrrolidine | Sponge of Baltic Sea (Germany) & Antarctic | *Lindgomyces LF327 & KF970* | Medium & inc. | *MRSE, SA, SEp* | Gram+ | / | Wu *et al.*, 2015 |
| 3. | Pyrrospirones C | Pyrrolidine | Wild crab, seaside rocks, China | *Penicillium sp. ZZ380* | Medium & inc. | *EC, MRSA* | Gram+ & Gram^−^ | / | Song *et al.*, 2018 |
| 4. | Pyrrospirones D | Pyrrolidine | Wild crab, seaside rocks, China | *Penicillium sp. ZZ380* | Medium & inc. | *EC, MRSA* | Gram+ & Gram^−^ | / | Song *et al.*, 2018 |
| 5. | Pyrrospirones E | Pyrrolidine | Wild crab, seaside rocks, China | *Penicillium sp. ZZ380* | Medium & inc. | *EC, MRSA* | Gram+ & Gram^−^ | / | Song *et al.*, 2018 |
| 6. | Pyrrospirones F | Pyrrolidine | Wild crab, seaside rocks, China | *Penicillium sp. ZZ380* | Medium & inc. | *EC, MRSA* | Gram+ & Gram^−^ | / | Song *et al.*, 2018 |
| 7. | Pyrrospirones I | Pyrrolidine | Wild crab, seaside rocks, China | *Penicillium sp. ZZ380* | Medium & inc. | *EC, MRSA* | Gram+ & Gram^−^ | / | Song *et al.*, 2018 |
| 8. | Penicipyrrodiether A | Pyrrolidine | Wild crab, seaside rocks, China | *Penicillium sp. ZZ380* | Medium & inc. | *EC, MRSA* | Gram+ & Gram^−^ | / | Song *et al*., 2018b |
| 9. | Penicipyrroether A | Pyrrolidine | Wild crab, seaside rocks, China | *Penicillium sp. ZZ380* | Medium & inc. | *EC, MRSA* | Gram+ & Gram^−^ | / | Song *et al*., 2019 |
| 10. | GKK1032C | Pyrrolidine | Mangrove plant, China | *Penicillium sp. CPCC 400817* | Inc. | *MRSA* | Gram+ | / | Qi *et al*., 2019 |
| 11. | Brocapyrrozin A | Pyrrolizidine | Mangrove plant, China | *Penicillium brocae MA-231* | Medium & dil. | *FO, SA* | F & Gram+ | / | Meng *et al*., 2017 |
| 12. | Brocapyrrozin B | Pyrrolizidine | Mangrove plant, China | *Penicillium brocae MA-231* | Medium & dil. | *FO* | F | / | Meng *et al*., 2017 |
| 13. | Penochalasin K | Indole | Mangrove plant, China | *Penicillium chrysogenum* | Medium & inc. | *CG, CM, PI, RS* | F | *che* (AM779763.1)& CHGG | Zhu *et al*., 2017 |
| 14. | 19-Hydroxypenitrem A | Diterpenoid indole | Marine red alga, China | *Aspergillus nidulans EN-330* | Inc. | *EC, ET, SA, VAn* | Gram+ & Gram^−^ | *ptm* (LC027936.1 & LC027937.1) | Zhang *et al*., 2015 |
| 15. | 6-Hydroxylpaspalinine | Diterpenoid indole | Sea anemone, China | *Penicillium sp. AS-79* | Medium & inc. | *VP* | Gram^−^ | *atm* (AY559849.2 & AM921700.1) | Hu *et al.*, 2017 |
| 16. | Penijanthine C | Diterpenoid indole | Sediment, China | *Penicillium janthinellum* | Medium & inc. | *VA, VAn, VP* | Gram^−^ | / | Guo *et al.*, 2019 |
| 17. | Penijanthine D | Diterpenoid indole | Sediment, China | *Penicillium janthinellum* | Medium & inc. | *VA, VAn, VP* | Gram^−^ | / | Guo *et al.*, 2019 |
| 18. | (3*R*,9*S*,12*R*,13*S*,17*S*,18*S*)-2-carbonyl-3-hydroxylemeniveol | Terpenoid indole | Marine mud, China | *Aspergillus versicolor ZZ761* | Inc. | *EC, CA* | F & Gram^−^ | *ptm*/*atm*? | Zhang *et al*., 2019 |
| 19. | Fumigatoside E | Quinazoline indole | Deep-sea sediments, Indian Ocean | *Aspergillus fumigatus SCSIO 41012* | Medium & inc. | *AB, FO, KP, SA* | F, Gram+ & Gram^−^ | Af12040-Af12080/*tqa*? | Limbadri *et al.*, 2018 |
| 20. | Fumigatoside F | Quinazoline indole | Deep-sea sediments, Indian Ocean | *Aspergillus fumigatus SCSIO 41012* | Medium & inc. | *AB* | Gram^−^ | Af12040-Af12080/*tqa*? | Limbadri *et al.*, 2018 |
| 21. | Oxysporizoline | Quinazoline | Mudflat, South Korea | *Fusarium oxysporum* | Medium & inc. | *MRSA* | Gram+ | / | Nenkep *et al.*, 2016 |
| 22. | Thielaviazoline | Quinazoline | Mudflat, South Korea | *Thielavia sp.* | Medium & PF | *MRSA* | Gram+ | / | Leutou *et al.*, 2016 |
| 23. | 2-(4-hydroxybenzyl)-4-(3-acetyl)quinazolin-one | Quinazoline | Seawater, China | *Aspergillus sydowii SW9* | Inc. | *EC, SA, SE, SP* | Gram+ & Gram^−^ | / | Liu *et al*., 2019 |
| 24. | 9-Hydroxy-3-methoxyviridicatin | Quinoline | Hydrovental crab, Taiwan | *Aspergillus versicolor XZ-4* | Medium & inc. | *EC* | Gram^−^ | *asq*, *pen* (KX528209.1) & *png* | Pan *et al*., 2017 |
| 25. | 7-Methoxycyclopeptin | Quinoline | Hydrovental crab, Taiwan | *Aspergillus versicolor XZ-4* | Medium & inc. | *EC* | Gram^−^ | *asq*, *pen* (KX528209.1) & *png* | Pan *et al*., 2017 |
| 26. | 7-Methoxycyclopenin | Quinoline | Hydrovental crab, Taiwan | *Aspergillus versicolor XZ-4* | Medium & inc. | *EC* | Gram^−^ | *asq*, *pen* (KX528209.1) & *png* | Pan *et al*., 2017 |
| 27. | Penicillatide B | Diketopiperazine | Red Sea tunicate, Egypt | *Penicillium sp.* | Medium & inc. | *CA, SA, VAn* | F, Gram+ & Gram^−^ | / | Youssef *et al.*, 2018 |
| 28. | Penicibrocazine B | Diketopiperazine | Mangrove plant, China | *Penicillium brocae MA-231* | Medium & inc. | *GG, SA* | F & Gram+ | / | Meng *et al.*, 2015 |
| 29. | Penicibrocazine C | Diketopiperazine | Mangrove plant, China | *Penicillium brocae MA-231* | Medium & inc. | *ML, SA* | Gram+ | / | Meng *et al.*, 2015 |
| 30. | Penicibrocazine D | Diketopiperazine | Mangrove plant, China | *Penicillium brocae MA-231* | Medium & inc. | *GG, SA* | F & Gram+ | / | Meng *et al.*, 2015 |
| 31. | Penicibrocazine E | Diketopiperazine | Mangrove plant, China | *Penicillium brocae MA-231* | Medium & inc. | *GG* | F | / | Meng *et al.*, 2015 |
| 32. | Brocazine G | Diketopiperazine | Mangrove plant, China | *Penicillium brocae MA-231* | OSMAC | *SA* | Gram+ | / | Meng *et al.*, 2016 |
| 33. | Spirobrocazine A | Diketopiperazine | Mangrove plant, China | *Penicillium brocae MA-231* | OSMAC | *EC, SA, VH* | Gram+ & Gram^−^ | / | Meng *et al.*, 2016 |
| 34. | Spirobrocazine C | Diketopiperazine | Mangrove plant, China | *Penicillium brocae MA-231* | OSMAC | *AH, EC, VH* | Gram^−^ | / | Meng *et al.*, 2016 |
| 35. | Acremolin B | Purine | Soil, Great Wall Station, Antarctic | *Aspergillus sydowii SP-1* | Medium & inc. | *MRSA, MRSE, SA, SE* | Gram+ | / | Li *et al*., 2018 |

Discovery Method

Medium & inc.: Nutrient optimization and incubation time

Medium & dil.: Nutrient optimization and dilution

Inc.: Incubation time only

Medium & PF: Nutrient optimization and precursor feed

OSMAC: One Strain MAny Compounds

Microorganism legend:

| AB | *Acinetobacter baumannii* (Gram^−^) | FO | *Fusarium oxysporum* (Fungus) | SA | *Staphylococcus aureus* (Gram^+^) |
| --- | --- | --- | --- | --- | --- |
| ABr | *Alternaria brassicae* (Fungus) | GG | *Gaeumannomyces graminis* (Fungus) | SE | *Salmonella enterica* (Gram^−^) |
| AH | *Aeromonas hydrophila* (Gram^−^) | KP | *Klebsiella pneumoniae* (Gram^−^) | SEp | *Staphylococcus epidermidis* (Gram^+^) |
| CA | *Candida albicans* (Fungus) | ML | *Micrococcus luteus* (Gram^+^) | SP | *Streptococcus pneumoniae* (Gram^+^) |
| CG | *Colletotrichum gloeosprioides* (Fungus) | MRSA | Multi-drug resistant *S. aureus* (Gram^+^) | VA | *Vibrio alginolyticus* (Gram^−^) |
| CM | *Colletotrichum musae* (Fungus) | MRSE | Methicillin-resistant *S. epidermidis* (Gram^+^) | VAn | *Vibrio anguillarum* (Gram^−^) |
| EC | *Escherichia coli* (Gram^−^) | PI | *Penicillium italicum* (Fungus) | VH | *Vibrio harveyi* (Gram^−^) |
| ET | *Edwardsiella tarda* (Gram^−^) | RS | *Rhizoctonia solani* (Fungus) | VP | *Vibrio parahemolyticus* (Gram^−^) |
